# Reply to: “Variables in the effect of land use on soil extrapore enzymatic activity and carbon stabilization” by Glenn (2020)

**DOI:** 10.1038/s41467-020-19901-8

**Published:** 2020-12-22

**Authors:** A. N. Kravchenko, A. K. Guber, B. S. Razavi, J. Koestel, M. Y. Quigley, G. P. Robertson, Y. Kuzyakov

**Affiliations:** 1 Department of Plant, Soil and Microbial Sciences, Michigan State University, East Lansing, MI 48824 USA; 2 DOE Great Lakes Bioenergy Research Center, Michigan State University, East Lansing, MI USA; 3 Department of Agricultural Soil Science, University of Göttingen, Göttingen, Germany; 4 Institute of plant nutrition and soil science, Kiel University, Kiel, Germany; 5 Swedish University of Agricultural Sciences, Uppsala, Sweden; 6 W. K. Kellogg Biological Station, Michigan State University, Hickory Corners, MI 49060 USA; 7 Institute of Physicochemical and Biological Problems in Soil Science, Pushchino, 142290 Russia; 8 RUDN University, Moscow, Russia

**Keywords:** Biogeochemistry, Environmental sciences

## Introduction

**Replying to** Glenn, M. *Nature Communications* https://doi.org/10.1038/s41467-020-19900-9 (2020)

We appreciate the comments by Glenn^1^ regarding our study of the plant system effects on soil pore characteristics and their implications for soil carbon storage (Kravchenko et al.^2^). We value the opportunity to respond and are happy to clarify the nature of the presented data and the design of our study, lack of clarity in which apparently generated Glenn’s^1^ concerns. We also would like to further clarify the mechanisms that underline the observed associations between pore sizes and enzyme activities, which were not articulated in detail in the original paper.

The first concern of Dr. Glenn is related to the data presented in Fig. 4a of the original paper. The presented results are not the absolute values of the enzyme activities, but relative activities standardized across each zymography map within each soil core. By construction, the average activity across each individual complete zymography map was set equal to zero. Hence, the averages for all systems across all pore sizes are also zeros, making any general statement regarding which system has higher/lower enzyme activity based on Fig. 4a data meaningless. The standardization was necessary to explore the distribution of enzyme activities associated with pores of specific sizes. The only meaningful comparisons using this data are the comparisons among the pore size groups within individual systems and this is what is reported in the study. From that perspective, the low values mean that the enzyme activities for particular pore size groups were close to the overall averages. Positive values indicated the prevalence of the above average values associated with certain pore size groups and negatives—below average values.

**Fig. 1:**
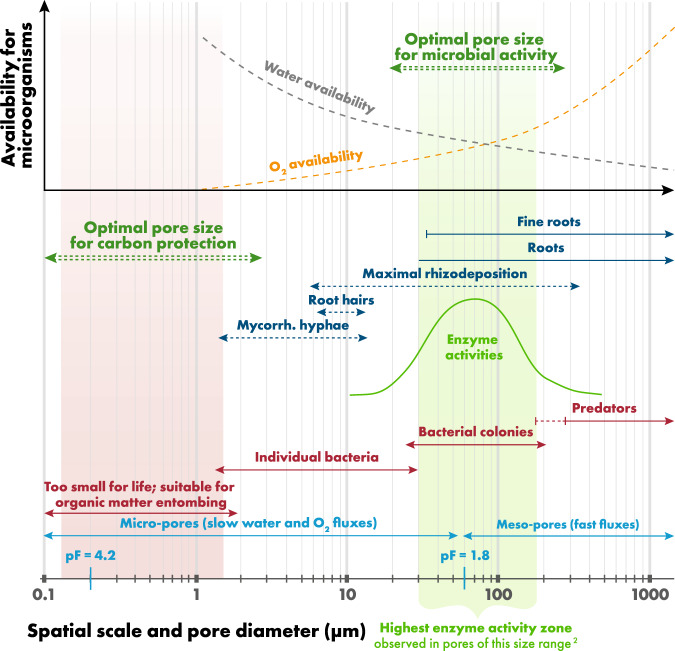
Intra-pore environmental conditions related to biological and enzyme activities as a function of pore size. Water and gas fluxes are very slow in few-micron-sized pores, increasing rapidly with increasing pore size (blue lines and text). Pores <1 µm diameter are too small for hosting microorganisms, and only single bacteria cells or fungal hyphae can be hosted within <10 µm pores. Pores with 10 to few-hundred-µm diameters accommodate fine plant roots and root hairs and are the locations of maximal rhizodeposition. This pore range is optimal for microorganisms due to plant inputs, and optimal water and O_2_ availabilities (top graph). This is the range of pores with the highest enzyme activities elucidated by zymography and µCT^2^ results of our study (shaded green). The pores of larger size are occupied by predators decreasing microbial density by grazing. Carbon sequestration is mainly taking place in the pores smaller than few micrometers by entombing: spatial exclusion from microbial and enzymatic activities (shaded red).

Association of the above-average enzyme activities with large pores in switchgrass but not in biodiverse systems is what formed the basis for our main conclusions. It is not the overall differences in microbial activities (those were similarly high in switchgrass and biodiverse systems (Fig. 1a in the original paper)), and not the overall differences in the enzyme activities (Suppl Fig. 4 in the original paper), but the fascinating differences in spatial patterns of the enzyme activities is what led to our conclusions.

Our experimental results are consistent with mechanistic understanding of the role that pores of various sizes play in soil functioning. While it is discussed in the original manuscript, here we further elucidate the relationships (Fig. 1). The finest pores (Ø < 1 µm) only minimally contribute to microbial activities because they are inaccessible to soil microorganisms due to size limitations. They experience only negligible influx of extracellular enzymes due to lack of diffusion of large enzyme molecules^3^. The water contained in the finest pores, while plentiful, is unavailable due to high matric potentials; and their O_2_ supply is limited (Fig. 1). However, these pores are excellent for storage of organic molecules enabling entombing and long-term carbon sequestration by spatial exclusion^4^. While pores in 1–10 µm Ø size range can harbor individual microorganisms, they do not provide enough space for microbial colonies^5^, not surprisingly having low microbial activity. Microbial colonies, crucial for C and N cycling, can be built within the pores with diameters at least a few tens of microns. The same pores are occupied by young fine roots which generate maximal rhizodeposition. The water supply in these pores is optimal and O_2_ is unlimited. The pores a few hundreds of microns in diameter are well accessible to soil animals grazing on bacteria and fungi. Moreover, these pores tend to drain and dry out the fastest, shortening the periods optimal for microbial activity.

Tendencies for higher enzyme activities in 30–150 µm pores in soils of low diversity systems observed in our study agree with the notion of pores of this size range forming an optimal habitat for microbial colonies, with plentiful nutrient supply via rhizodeposition and water influxes, optimal balance between air and water regimes, and low predation. Consequently, they experience high microbial densities and, hence, high enzyme activities. Last but not least, very fine roots populating these pores are another important source of extracellular enzyme release.

In the diverse plant systems, high abundance, connectivity, and wide spread of such pores through the soil matrix led to ubiquitous presence of microorganisms, fine roots, and enzymes. While in the low diversity systems, where such pores were not so prevalent and sparsely placed, their positive contribution to microbial activity and resultant enzyme patterns was clearly pronounced.

The second concern of Dr. Glenn is that the observed differences in soil properties cannot be attributed to plant diversity but are generated by other sources, such as differences in inherent soil characteristics, faunal and groundwater movements, and historical management differences. As described in the original paper, the experimental design of our field study is a randomized complete block with five replications. At the study initiation in 2007, the blocks were laid out to minimize spatial variability patterns in inherent soil and topographical characteristics, and the plant system treatments were randomly assigned to the experimental plots within each block. Thus, it can be safely concluded that the developed differences in the soil characteristics were caused by the plant system effects, of which plant diversity is the most pronounced feature. In fact, the only explanatory variable which can be used to generate cause-and-effect conclusions in this study is plant system/plant diversity. The influence of the other inherent factors (mineral content, depth of surface and subsurface horizons, historical management practices) can be considered as equally affecting all studied plant systems, due to the replicated blocked experimental design with random treatment assignment.

We feel strongly that it is not enough to stop at the conclusion that plant diversity causes differences in soil pore characteristics. Plant diversity manifests its influence on soil pore structure through a variety of physical, chemical, and biological controls. It influences soil organic matter levels, quality of soil carbon inputs, macro and mesofauna and their movements. Those factors were most likely among the key agents through which differences in plant diversity created the differences observed here in soil pore size distributions and enzyme localization patterns between plant systems. It is of outmost importance to focus future research efforts on determining drivers operating in diverse plant communities that shape soil pore architecture. While we can speculate on potential contributions of uncontrolled variables, the only scientifically warranted conclusion from our study is that the plant systems with different diversity levels caused the observed differences.

### Reporting summary

Further information on research design is available in the Nature Research Reporting Summary linked to this article.

## Supplementary information

Reporting Summary

## Data Availability

No new data were generated for this response. Data collected for the original study by Kravchenko et al.^2^ are provided as a Source Data file and will be preserved in the KBS LTER database available at http://lter.kbs.msu.edu/datatables.

## Code availability

No new codes were generated for this response. Data and image analyses codes for the original study by Kravchenko et al.^2^ are available from the authors upon request.

## References

[1] Glenn, M. Matters arising: Variables in the effect of crop field biodiversity on soil extrapore enzymatic activity and carbon stabilization. *Nat. Commun.* In press. 10.1038/s41467-020-19900-9 (2020). 10.1038/s41467-020-19900-9 PMC7755900 33353929

[2] Kravchenko, A. N. et al. Microbial spatial footprint as a driver of soil carbon stabilization. *Nat. Commun.* **10**, 3121 (2019). 10.1038/s41467-019-11057-4 PMC6635512 31311923

[3] Guber A (2018). Quantitative soil zymography: mechanisms, processes of substrate and enzyme diffusion in porous media. Soil Biol. Biochem..

[4] Liang, C., Schimel, J. P. & Jastrow, J. D. The importance of anabolism in microbial control over soil carbon storage. *Nat. Microbiol.* **2**, 17105 (2017). 10.1038/nmicrobiol.2017.105 28741607

[5] Kuzyakov Y, Blagodatskaya E (2015). Microbial hotspots and hot moments in soil: concept & review. Soil Biol. Biochem..

